# Exploring an Uncommon Presentation of Kikuchi-Fujimoto Disease: Case Insights

**DOI:** 10.7759/cureus.67338

**Published:** 2024-08-20

**Authors:** Bollineni S Parda, Babaji Ghewade, Ulhas Jadhav, Pankaj Wagh, Vivek D Alone

**Affiliations:** 1 Respiratory Medicine, Jawaharlal Nehru Medical College, Datta Meghe Institute of Higher Education and Research, Wardha, IND

**Keywords:** benign disease, lymph node, corticosteroid treatment, pleural effusion, inflammation

## Abstract

Kikuchi-Fujimoto disease (KFD) is a self-limiting, benign illness that is typified by cervical lymphadenopathy, typically accompanied by a low fever and night sweats. Loss of weight, nausea, vomiting, and sore throat are fewer common symptoms. KFD can have an acute or subacute start, and it usually develops over two to three weeks. Although viral aetiology is still a concept that needs further research, the clinical, histological, and immunohistochemical aspects seem to support it. Since specific diagnostic laboratory tests are not available, the diagnosis is frequently established by excising a sample of the affected lymph nodes. This case study features a 63-year-old male patient who first complained of fever and dyspnoea. Upon further investigation, the patient's condition was determined to be Kikuchi-Fujimoto disease, which was treated appropriately.

## Introduction

Kikuchi-Fujimoto disease (KFD) is an uncommon inflammatory illness that primarily affects younger people and paediatric patients. The typical presentation, which can range from acute to subacute, is marked by painful, tender lymphadenopathy together with systemic symptoms such as fever, malaise, weight loss, arthralgias, and other cutaneous indications [1].

There is much speculation about the aetiology of KFD. A viral or autoimmune cause has been suggested. The role of Epstein-Barr virus as well as other viruses (human herpesvirus 6 (HHV6), HHV8, parvovirus B19) in the pathogenesis of KFD remains controversial and not convincingly demonstrated. Despite this, Hudnall et al. have reported that clinical manifestations such as upper respiratory prodrome, atypical lymphocytosis, and specific histopathological characteristics may indicate the possibility of a viral infection [2].

KFD has additionally been observed in individuals who are HIV- and human T-lymphotropic virus (HTLV-1)-positive. However, tubular reticular structures have been found in the cytoplasm of activated lymphocytes and histiocytes in individuals with KFD, according to electron microscopic investigations. A conclusive diagnosis requires an excisional lymph node biopsy, which reveals a neutrophil and eosinophil deficit. Histiocytes positive for myeloperoxidase and cluster of differentiation 68 (CD68) and a negligible amount of B cells will all be seen by immunohistochemistry. Because fine-needle aspiration obtains a limited amount of tissue, it is usually insufficient for the confirmation of the diagnosis. It is crucial to distinguish KFD from lymphomas and infectious aetiologies. Cultures and serological testing can provide more evidence in support of the histologic diagnosis [3].

## Case presentation

**Figure 1:**
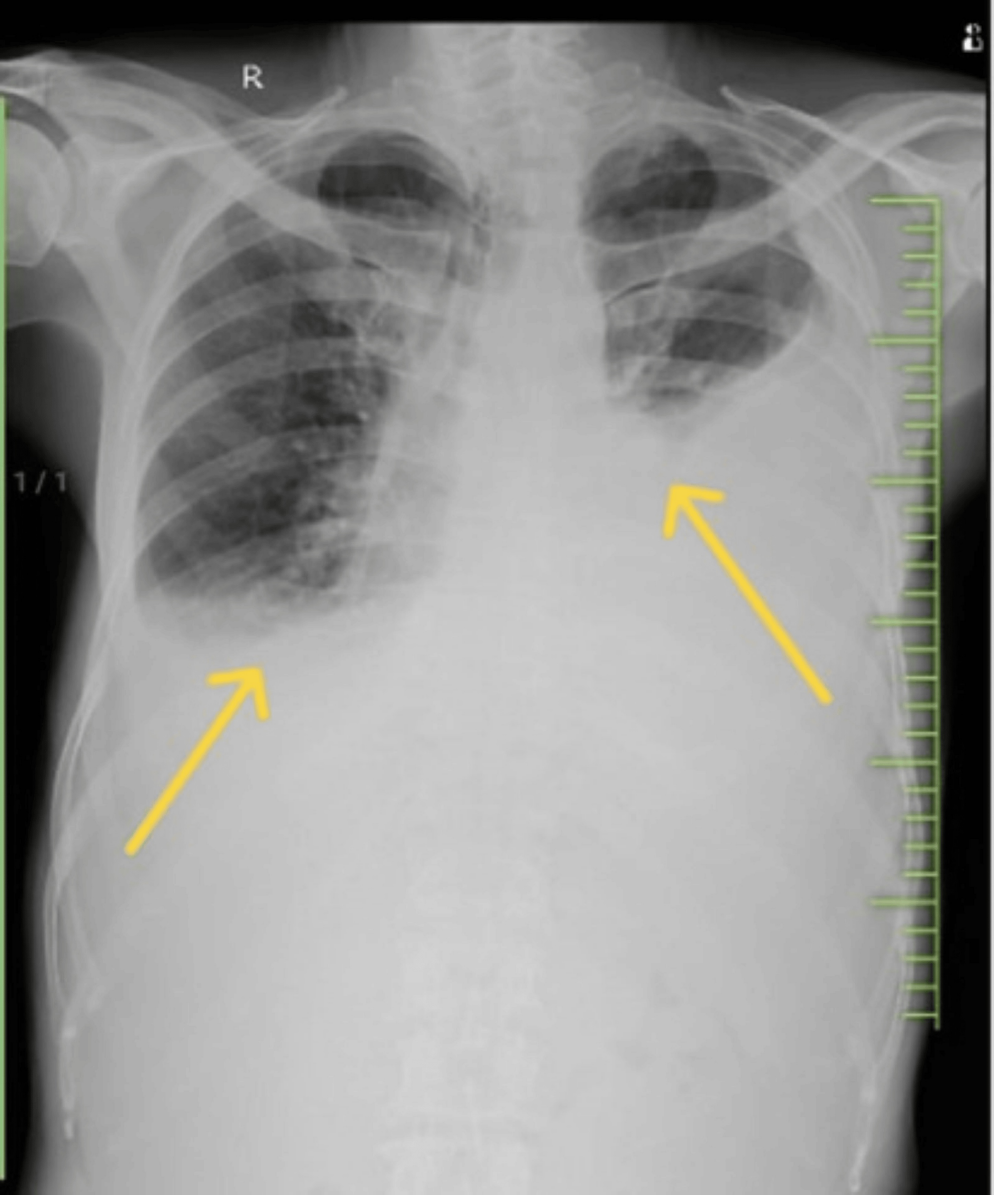
Chest X-ray showing bilateral pleural effusion

**Figure 2:**
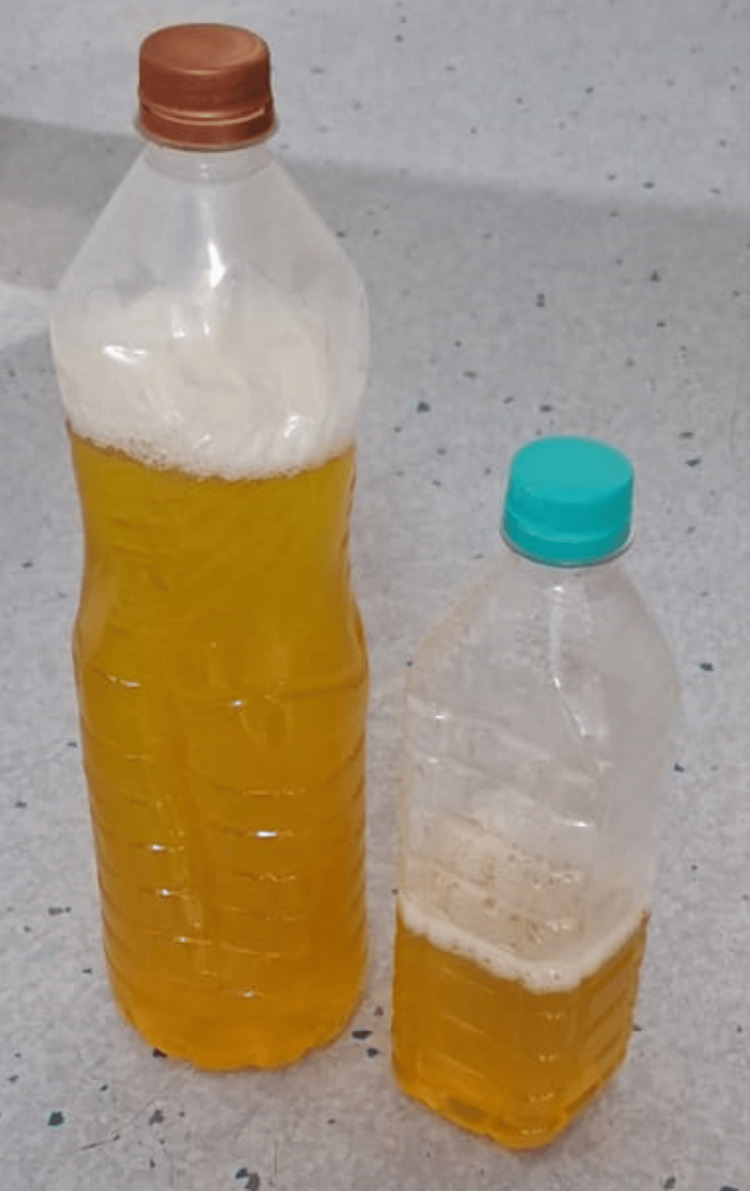
Straw-coloured pleural fluid removed through ultrasonography (USG)-guided thoracocentesis

**Figure 3:**
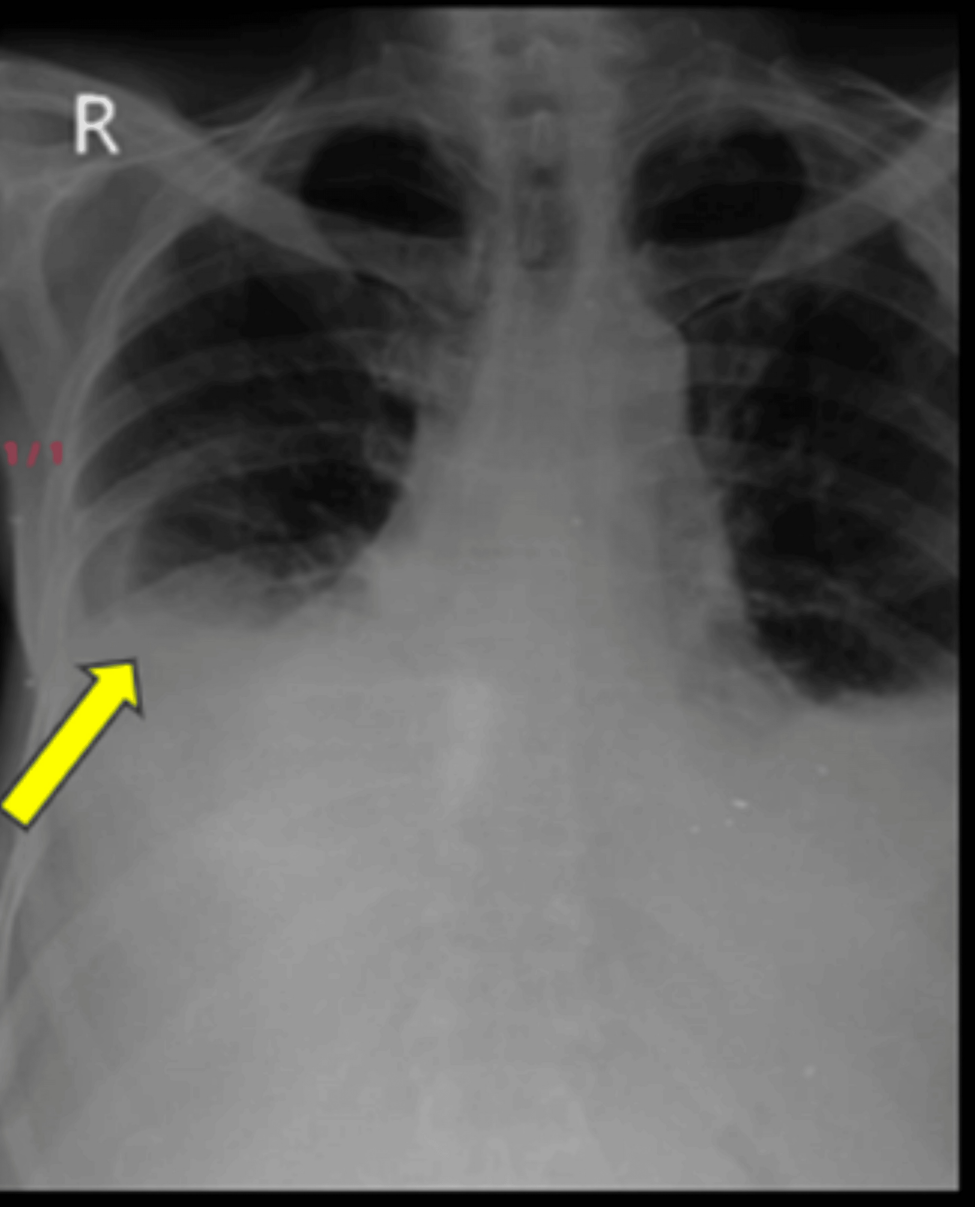
Chest X-ray (posterior-anterior (PA)) view after right-side thoracocentesis

**Figure 4:**
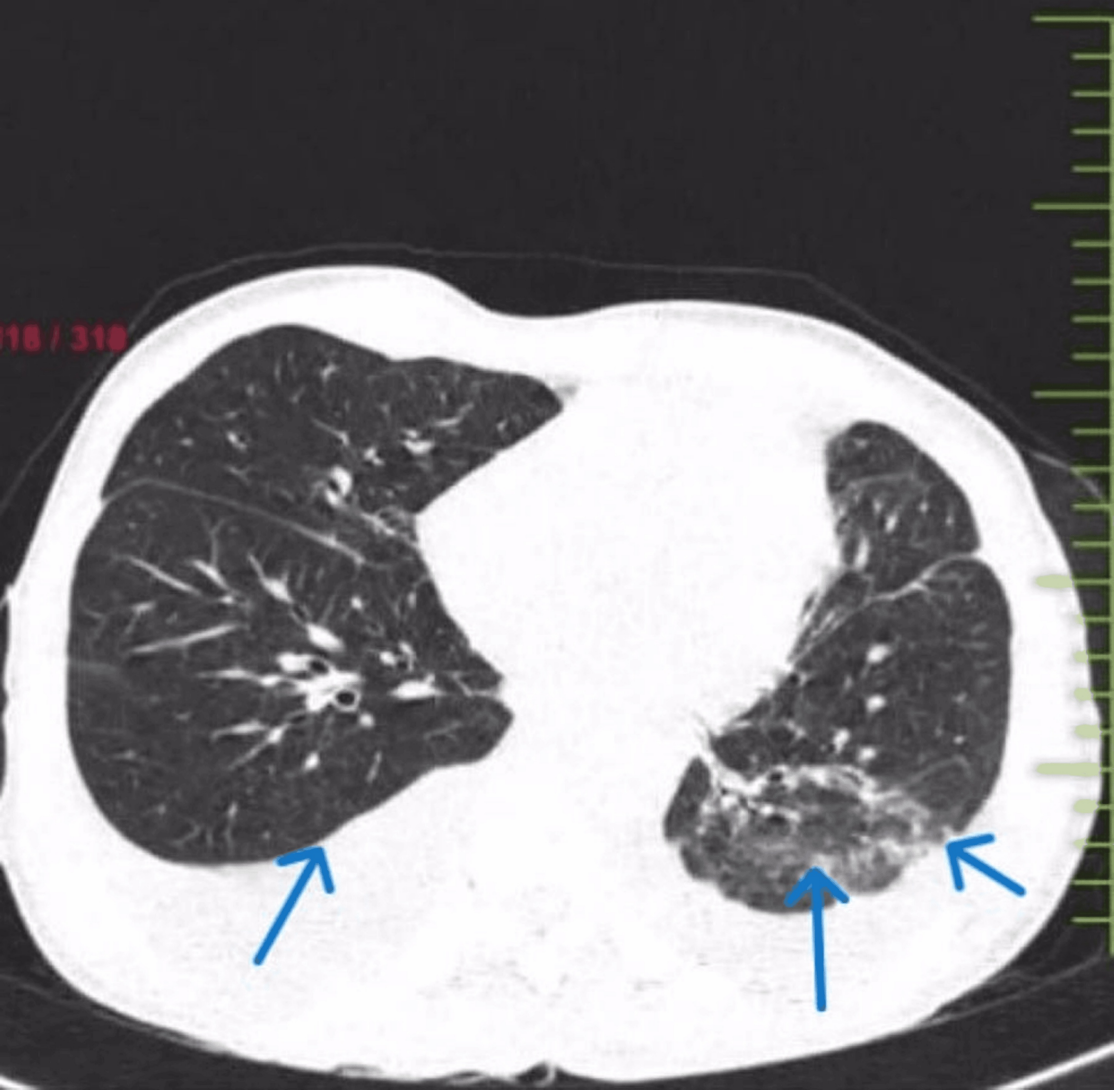
High-resolution computed tomograph (HRCT) of the thorax showing subcutaneous emphysema on the right side with bilateral pleural effusion with sub-segmental atelectasis and patchy areas of consolidation in lateral and posterior segments of the left lower lobe.

A 63-year-old male patient presented to the respiratory medicine department with the chief complaints of fever on and off for 15 days and dry cough associated with dyspnea on exertion for seven days. The patient also had a history of loss of appetite and loss of weight of 5-6 kg in two months. The patient had a known case of bilateral recurrent pleural effusion. There was no history of associated chest pain, hemoptysis, pleural tuberculosis or any comorbidities. A general examination revealed pallor and clubbing. On the examination of vitals, tachypnea and hypoxia were noted. A respiratory system examination revealed bilaterally reduced breathing sounds. The findings of lab investigations are given in Table 1. Chest X-ray (Figure 1) was done, which was suggestive of bilateral pleural effusion on the right side more than the left for which therapeutic tapping was done, and 1500 ml fluid was drained (Figures 2, 3). Lymph node examination was done, which was suggestive of multiple palpable lymph nodes predominantly (left posterior auricular, left posterior cervical and right occipital group of lymph nodes). Acid-fast bacillus (AFB) test and Trunet assay of the sputum and pleural fluid were found to be negative. Pleural fluid investigations suggested exudative pleural effusion with low adenosine deaminase (ADA) according to light criteria. Biopsy showed lymph nodes with follicular hyperplasia (lymphoid) and focal, circumscribed, paracortical necrotizing lesions composed of abundant karyorrhectic debris, and a collection of large mononuclear cell bronchoalveolar lavage (BAL) (culture and sensitivity) showed no growth. A high-resolution computed tomography (HRCT) of the thorax was suggestive of bilateral pleural effusion with sub-segmental atelectasis with bilateral patchy ground-glass opacifications (GGOs) and consolidation (Figure 4).

**Table 1:** Laboratory findings Hb: haemoglobin; RBC: red blood cells; WBC: white blood cells; ALT (SGPT): alanine aminotransferase (serum glutamic-pyruvic transaminase); AST (SGOT): aspartate aminotransferase (serum glutamic oxaloacetic transaminase)

Test	Patient's value	Reference value
Hb	10.4	11-15 mg/dl
Total RBC count	5.96	3.8-5.8 million/cumm
WBC	12700	4000-11000/cumm
Total platelet count	2.69	150,000-400,000/cumm
Adenosine deaminase	16.24	18.46-27.50 U/L
Lactate dehydrogenase	258	<480 U/L
Urea	14	1.7-8.3 mmol/L
Creatinine	0.6	62-106 mmol/L
Sodium	137	137-147 mmol/L
Potassium	4.4	3.5-5.3 mmol/L
Alkaline phosphatase	107	44-147 IU/L
ALT (SGPT)	16	7-56 IU/L
AST (SGOT)	34	8-33 U/L
Total protein	5.4	6.7-8.6 g/dl
Albumin	3.1	3.5 -5.5 g/dl
Globulin	2.3	2.0-3.5 g/dl

Duplex colour doppler of the bilateral lower limb (arterial and venous) was found to be normal and no deep vein thrombosis was found.

**Figure 5:**
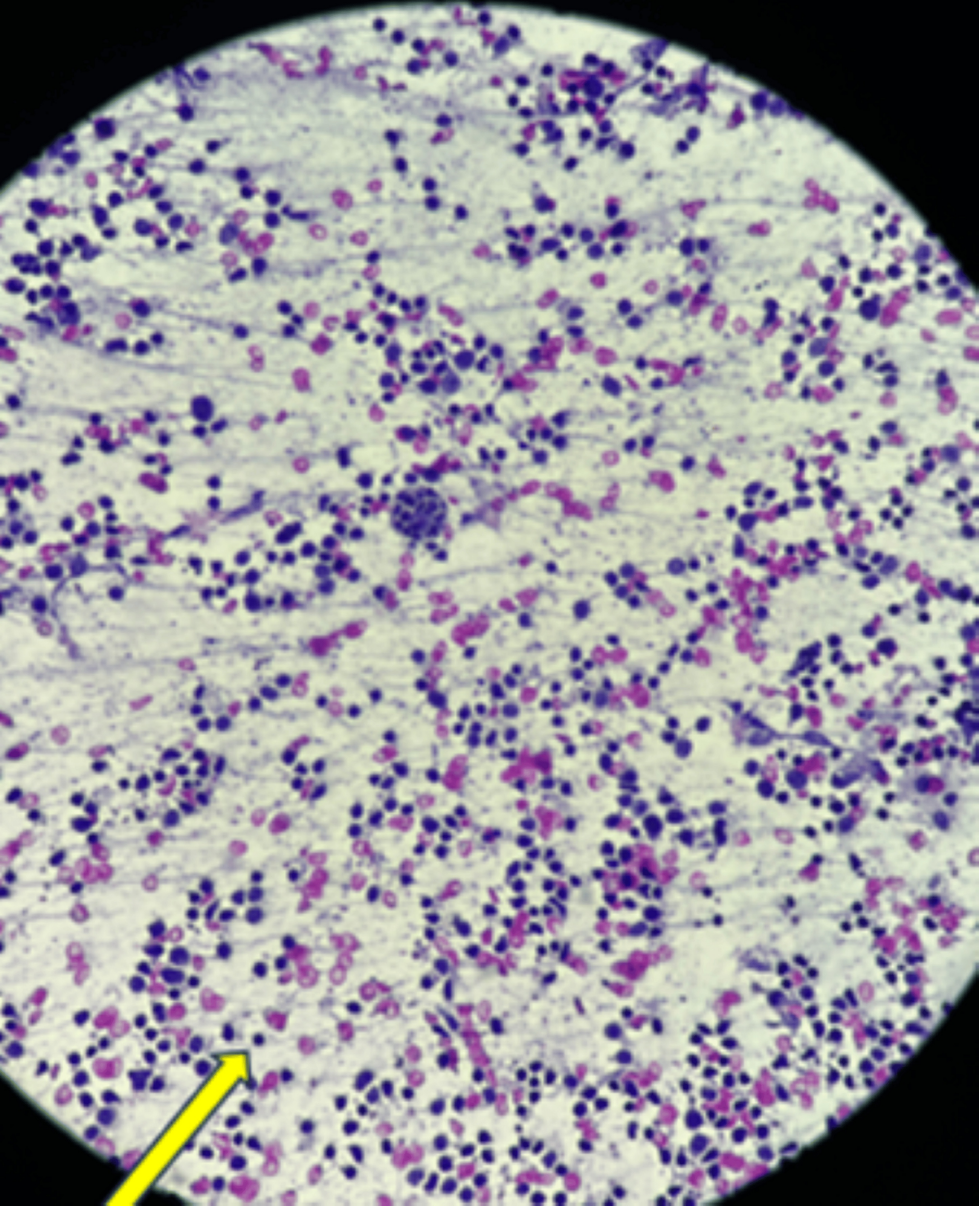
The section studied showed abundant macrophages, lymphocytes and emperipolesis

**Figure 6:**
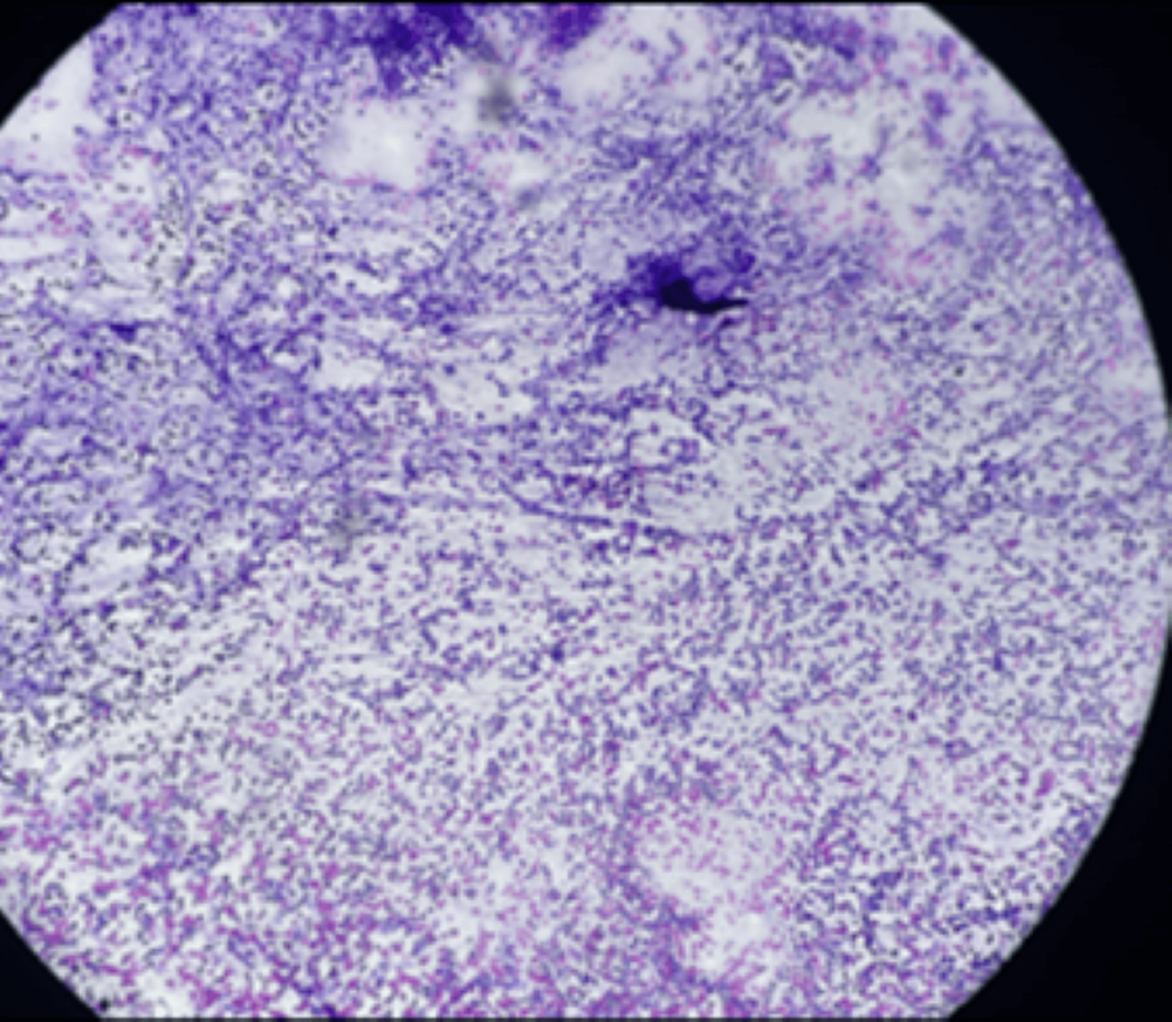
Section studied by lymph node fine-needle aspiration cytology (FNAC) shows follicular cells with abundant immunoblasts, lymphocytes and cluster of macrophages. Reactive hyperplasia with necrosis

Ultrasonography (USG)-guided cervical lymph node fine-needle aspiration cytology (FNAC) was done and the histopathology reports were suggestive of reactive lymphoid hyperplasia with necrotic changes (Figures 5, 6), which was diagnosed to be Kikuchi-Fujimoto disease for which the patient was started on corticosteroids. The patient was discharged when symptomatically better.

## Discussion

KFD is an incredibly unusual condition with an acute or subacute start that develops over two to three weeks. It is nearly always accompanied by cervical lymphadenopathy, which consists of sore lymph nodes primarily involving the posterior cervical triangle. Usually, KFD resolves on its own in three to four months. Recurrence rates of 3-4% have been documented, which is low but conceivable [4].

In a small percentage of people, systemic lupus erythematosus (SLE) may develop years later. There appears to be little danger to family members of the patients with KFD with regard to the disease. It is important to use symptomatic treatments to relieve the upsetting local and systemic complaints. The use of analgesics, antipyretics, and nonsteroidal anti-inflammatory drugs (NSAIDs) can help reduce fever and soreness in the lymph nodes. There are no established diagnostic standards for KFD because of its uncommon nature and inconsistent presentation. Clinicians must first rule out other possible causes of lymphadenopathy, such as infectious, neoplastic, and autoimmune origins, in order to make a diagnosis. Although its effectiveness is questionable, the administration of corticosteroids has been advised in cases of severe extra-nodal or widespread KFD. To perform a diagnostic excisional lymph node biopsy, surgical consultation may be necessary. To rule out the development of SLE, patients with KFD need to undergo a comprehensive survey and ongoing follow-up for a few years. After a definitive diagnosis, the cervical lymphadenopathy has a benign course and seems to go away on its own within one to six months [5,6].

## Conclusions

This case report highlights a rare presentation of Kikuchi-Fujimoto Disease (KFD) in a 63-year-old male, characterized by bilateral recurrent pleural effusions and significant respiratory symptoms. The final diagnosis of KFD was established through ultrasound-guided FNAC of the cervical lymph nodes, revealing reactive lymphoid hyperplasia with necrotic changes. Treatment for KFD generally involves supportive care, with NSAIDs and glucocorticoids providing significant symptomatic relief. This case emphasizes the importance of including KFD in the differential diagnosis for patients presenting with lymphadenopathy, even in the presence of symptoms like pleural effusion. The histopathological evaluation was crucial in reaching an accurate diagnosis and directing appropriate treatment strategies. The patient's favourable response to corticosteroid therapy and subsequent discharge in a stable condition emphasizes the potential for positive outcomes with appropriate recognition and management of this rare disorder. Understanding the clinical and histopathological features of KFD and its treatment options can help clinicians provide optimal care and avoid unnecessary procedures.
